# NF-κBp65 and Expression of Its Pro-Inflammatory Target Genes Are Upregulated in the Subcutaneous Adipose Tissue of Cachectic Cancer Patients

**DOI:** 10.3390/nu7064465

**Published:** 2015-06-04

**Authors:** Rodolfo Gonzalez Camargo, Daniela Mendes dos Reis Riccardi, Henrique Quintas Teixeira Ribeiro, Luiz Carlos Carnevali, Emidio Marques de Matos-Neto, Lucas Enjiu, Rodrigo Xavier Neves, Joanna Darck Carola Correia Lima, Raquel Galvão Figuerêdo, Paulo Sérgio Martins de Alcântara, Linda Maximiano, José Otoch, Miguel Luiz Batista, Gerhard Püschel, Marilia Seelaender

**Affiliations:** 1 Cancer Metabolism Research Group, Institute of Biomedical Sciences, University of Sao Paulo, Av. Prof. Lineu Prestes, 1524—Cidade Universitária, Sao Paulo, 05508-000, Brazil; E-Mails: dariccardi@hotmail.com (D.M.R.R.); henriqueribeiro@hotmail.com (H.Q.T.R.); lucarjr78@hotmail.com (L.C.C.); emidiomatos@gmail.com (E.M.M.-N.); enjiu84@gmail.com (L.E.); digo_e.d@hotmail.com (R.X.N.); joana.carola14@gmail.com (J.D.C.C.L.); raquel.galfig@gmail.com (R.G.F.); seelaend@icb.usp.br (M.S.); 2 Department of Clinical Surgery, University of Sao Paulo, Av. Prof. Lineu Prestes, 2565—Cidade Universitária, São Paulo, 05508-000, Brazil; E-Mails: palcantara@usp.br (P.S.M.A.); linda@usp.br (L.M.); pinhata@usp.br (J.O.); 3 Biotechnology Group, Laboratory of Adipose Tissue Biology, University of Mogi das Cruzes, Mogi das Cruzes, Sao Paulo, 05508-100, Brazil; E-Mail: migueljr4@me.com; 4 Department of Nutritional Biochemistry, University of Potsdam, Potsdam, 14558, Germany, E-Mail: gpuesche@uni-potsdam.de

**Keywords:** cancer cachexia, inflammation, white adipose tissue, NF-κB, IκB

## Abstract

Cancer cachexia, of which the most notable symptom is severe and rapid weight loss, is present in the majority of patients with advanced cancer. Inflammatory mediators play an important role in the development of cachexia, envisaged as a chronic inflammatory syndrome. The white adipose tissue (WAT) is one of the first compartments affected in cancer cachexia and suffers a high rate of lipolysis. It secretes several cytokines capable of directly regulating intermediate metabolism. A common pathway in the regulation of the expression of pro-inflammatory cytokines in WAT is the activation of the nuclear transcription factor kappa-B (NF-κB). We have examined the gene expression of the subunits NF-κBp65 and NF-κBp50, as well as NF-κBp65 and NF-κBp50 binding, the gene expression of pro-inflammatory mediators under NF-κB control (IL-1β, IL-6, INF-γ, TNF-α, MCP-1), and its inhibitory protein, nuclear factor of kappa light polypeptide gene enhancer in B-cells inhibitor, alpha (IκB-α). The observational study involved 35 patients (control group, *n* = 12 and cancer group, *n* = 23, further divided into cachectic and non-cachectic). NF-κBp65 and its target genes expression (TNF-α, IL-1β, MCP-1 and IκB-α) were significantly higher in cachectic cancer patients. Moreover, NF-κBp65 gene expression correlated positively with the expression of its target genes. The results strongly suggest that the NF-κB pathway plays a role in the promotion of WAT inflammation during cachexia.

## 1. Introduction

Cancer cachexia is mainly characterized by involuntary weight loss. This syndrome is present in around fifty percent of all cancer patients and may be found in more than two thirds of those in the advanced stage of the disease [1]. It represents the direct cause of at least twenty to forty percent of all deaths associated with cancer [2]. The etiology of cachexia is extremely complex and the syndrome compromises survival and quality of life [3]. It is currently accepted that systemic inflammation plays a major role in the plethora of alterations that characterize cachexia [4]. Indeed, high concentration of inflammatory cytokines is reported in the plasma and tissues of both animal models and patients [5]. Yet, one question remains unclear: What elements trigger and maintain cachexia-related systemic inflammation? We have previously provided evidence that the white adipose tissue is a potential contributor to the maintenance of systemic inflammation in cachexia, as it secretes several inflammatory cytokines and adipokines [4,6,7]. These factors directly regulate several functions related with metabolism, body composition, activity of the complement system and vascular homeostasis [8]. Among these adipose-derived factors, several pro-inflammatory and anti-inflammatory mediators are described, including tumor necrosis factor alpha (TNF-α), interleukin 1 beta (IL-1β), interleukin-6 (IL-6) and monocyte chemoattractant protein 1 (MCP-1) [9]. Therefore, besides being profoundly affected by cachexia [10], the adipose tissue may play an important role in its etiology.

A central step in the control of the cellular expression of pro-inflammatory cytokines is the activation in cells of the nuclear transcription factor kappa B (NF-κB), which induces the transcription of most genes related with inflammation, including the so called ‘classic cachectic cytokines’ TNF-α, IL-6, IL-1β, interferon gamma (INF-γ) and of chemokines such as MCP-1. This pathway also induces nitric oxide synthase (iNOS), as well as the expression of adhesion molecules [11]. A large body of evidence indicates a link between inflammation promoted by the activation of this transcription factor and cancer (with regard to tumor progression) and it has been shown that inhibition of NF-κB activation markedly affects cachexia [12]. Thus, NF-κB is considered a target for cancer treatment [13]. Furthermore, extensive lipolysis in white adipose tissue seems to be related with TNF-α action through the activation of the NF-κB signaling pathway, as demonstrated in cultured adipocytes [14].

The functional form of the molecule of NF-κB consists of dimers (homo-or heterodimers) [14], Different combinations of NF-κB subunits present different functions in the regulation of the immune response. The transcription of pro-inflammatory genes in the NF-κB classical signaling pathway is regulated by the heterodimer NF-κBp65-p50, while the homodimer NF-κBp50-p50 has been described as anti-inflammatory, repressing the expression of several pro-inflammatory molecules due to the absence of its COOH-terminal transactivation domain [15]. The most studied heterodimers are NF-κBp65/NF-κBp50 (NF-κBp65-p50), and NF-κBp52/RelB (NF-κBp52-RelB) [12]. The vast majority of the studies on inflammation focus on the heterodimer NF-κBp65-p50, due to its unequivocal inflammatory function. Given the potential role of the white adipose tissue to maintain systemic inflammation in cancer cachexia, and the high circulating levels of cytokines that are characteristic of the syndrome, we performed an observational study in which we examined, for the first time, the correlation of the dimer NF-κBp65-p50 with the induction of gene expression of pro-inflammatory cytokines and chemokines in the subcutaneous adipose tissue of cachectic cancer patients, as compared with non-cachectic.

## 2. Experimental Section

### 2.1. Patient Recruitment

Patients (*n* = 35) were recruited between July 2011 and January 2013 at the University Hospital of the University of São Paulo. The recruitment was conducted by the hospital personnel and consisted in selecting patients engaged in the treatment of hernia (control group (*N*), *n* = 12) and cancer, further divided in non-cachectic (T), *n* = 11 and cachectic [16], *n* = 12. The project was approved by the University of São Paulo Biomedical Sciences Institute Ethics Committee (1004/CEP), and by the University Hospital Ethics Committee (CEP-HU/USP: 752/07). The inclusion criteria were: not having received prior anticancer or anti-inflammatory treatment, and willingness to participate. The exclusion criteria were: liver failure, renal failure, AIDS, inflammatory diseases of the bowel and autoimmune disorders. After the selection, anthropometric measurements were obtained (height, weight) and the patients were interviewed with a quality of life questionnaire validated for Portuguese (EORTC QLQ-C30) [17,18], which addresses three clusters that compose quality of life: Functionality (physical, cognitive, emotional and social), Symptomatic (fatigue, pain, nausea and vomiting) and Global health. The cancer patients groups division was based on “Cachexia a new definition” [16], in which cachexia is diagnosed in patients with involuntary weight loss of at least 5% in the past 12 months or BMI <20 kg/m^2^, plus at least three of the five following criteria: decreased muscle strength, fatigue, anorexia, low fat-free mass index and abnormal biochemistry (increased circulated inflammatory markers as IL-6 >4.0 pg/mL or C-Reactive Protein (CRP) >5.0 mg/L, anemia (Hb < 12 g/dL) or low serum albumin (<3.2 g/dL). The non-cachectic cancer group was composed of patients under cancer treatment that did not fulfill the mentioned criteria. A full written consent form was obtained from each patient.

### 2.2. Clinical and Biochemical Parameters Assessment

Height and weight were determined and approximately 10mL of blood collected on the interview day previous to surgery. The samples were then centrifuged and serum was collected and frozen at −80 °C for further analysis. The serum measurements (CRP, Albumin) were performed with the commercial kit (Turbiquest plus (Cat# 331) ultrasensitive CRP and Albumin (Cat#19)) from Labstest, Lagoa Santa, MG, Brazil. Haemoglobin measurements were performed by the University Hospital laboratory (Cidade universitária, São Paulo, Brazil).

### 2.3. Adipose Tissue Biopsies

Approximately one gram of subcutaneous white adipose tissue was collected during surgery. Tissue samples were rapidly divided in two tubes: The first with 1 mL of Trizol^®^ for subsequent total RNA extraction and Quantitative real-time PCR (qPCR) experiments, and the second with 20 mL of PBS 1 X with 5% of phosphatase inhibitor for subsequent ELISA binding assay experiments. This procedure presented a minimal degree of risk, and did not interfere with the standard surgery procedure or with anesthesia.

### 2.4. Gene Expression

Total RNA was isolated using the Trizol^®^ Reagent according to the manufacturer’s instructions. Total RNA concentrations were quantified using the Biomate 3 spectrophotometer (Thermo Fisher Scientific Inc., Waltham, MA, USA). Complementary DNA synthesis was carried out using the high capacity cDNA reverse transcription kit (Life Technologies, Grand Island, NY, USA), which consisted of an assay mix containing 1 μg total RNA, 2 μL 10× RT Buffer, 0.8 μL 25× dNTP mix (100 mM), 2 μL 10× Random primers, 1 μL MultiScribe™ Reverse Transcriptase and 4.2 μL of nuclease-free water in a final volume of 20 μL. The thermal cycler conditions were: 25 °C for 10 min, then 37 °C for 120 min followed by 85 °C for 5 min. Then, 20 ng of cDNA were mixed with 2× SYBR Green fast PCR master mix—and primers (Table 1) (Life Technologies, Grand Island, NY, USA)—in a final volume of 10 μL for qPCR, performed in the Quantstudio 12K Real Time Systems (Life Technologies, Grand Island, NY, USA).The mRNA levels were determined by the comparative Ct method. For each sample, a ΔCt value was obtained by subtracting RPL-27 values from those of the gene of interest. The average ΔCt value of the control group was then, subtracted from the sample to derive a −ΔΔCt value. The expression of each gene was evaluated by 2^−ΔΔCt^, according to Livak *et al*. 2001 [19].

**Table 1:** Primer sequences used in the qPCR experiments.

Gene	Sense (5′–3′)	Antisense (5′–3′)
**RPL-27 (NM_000988.3)**	CCGAAATGGGCAAGTTCAT	CCATCATCAATGTTCTTCACGA
**NF-κBp65 (NM_021975.3)**	CCTGGAGCAGGCTATCAGTC	ATGGGATGAGAAAGGACAGG
**NF-κBp50 (NM_003998.3)**	CATCCCATGGTGGACTACCT	TGGGTCCAGCAGTTACAGTG
**IκB-α (NM_020529.2)**	CTCCGAGACTTTCGAGGAAATAC	GCCATTGTAGTTGGTAGCCTTCA
**IL-1β (NM_000576.2)**	AGCCAATCTTCATTGCTCAAGT	AGTCATCCTCATTGCCACTGT
**IL-6 (NM_000600.3)**	CAGCCCTGAGAAAGGAGACAT	AGCCATCTTTGGAAGGTTCA
**TNF-α (NM_000594.3)**	CTCTCTCCCCTGGAAAGGAC	ATCACTCCAAAGTGCAGCAG
**INF-γ (NM_000619.2)**	TGGAAAGAGGAGAGTGACAGAA	TGGAAAGAGGAGAGTGACAGAA
**MCP-1 (NM_002982.3)**	TCAGCCAGATGCAATCAATG	ACACTTGCTGCTGGTGATTCT

### 2.5. NF-κB Binding Assay

Subcutaneous adipose tissue protein extraction was carried out employing the Active Motif^®^ Nuclear extract kit (Active Motif^®^, Carlsbad, CA, USA) according to the manufacturer’s protocol. Total protein was assessed with the commercial Pierce BCA protein assay kit (Life Technologies, Grand Island, NY, USA), according to the manufacturer’s protocol. Western blot was performed to verify the efficacy of nuclear protein extraction as described below:

Samples were boiled at 95 °C for 5 min in SDS-mercaptoethanol sample buffer. Then, were centrifuged for 5 min at 12,000× *g*. Equal amounts of protein (20 μg per sample) were separated in the NuPAGE^®^ Novex^®^ 4%–12% Bis-Tris protein gel (Catalog # NP00336BOX) (Life Technologies, Grand Island, NY, USA) and then transferred to a PVDF membrane. After blocking with 5% non-fat milk in Tris buffered saline Tween 20 (TBS-Tween 0.1%) for 1 h at room temperature, membranes were washed three times with TBS-Tween 0.1% for 10 min and then incubated overnight with primary antibodies at 4 °C. The Primary antibodies against Lamin A 1:500 (Catalog # sc-20680; Lamin A antibody H-102) and β-Tubulin 1:1000 (Catalog # sc-9104 β-Tubulin antibody H-235) were obtained from Santa Cruz Biotechnology Inc. (Santa Cruz, CA, USA). After three washes on the next day, the membranes were incubated with anti-rabbit IgG secondary antibody (1:5000) for two hours. The membranes were then incubated with ECL-Plus chemiluminescent detection HRP reagents (Bio-rad, Hercules, California, USA). Immunoreactive bands were visualized using the ImageQuant LAS 4000 (GE, Fairfield, CT, USA).

The binding assay was performed employing the NF-κB Family Transam transcription factor assay kit^®^ (Active Motif^®^, Carlsbad, CA, USA), according to the manufacturer’s protocol which consisted of a NF-κB binding sequence (5′-GGGACTTTCC-3′) immobilized in each of the 96-well plate used in the assay. The protein extract (20 μg) was then pipetted in each well and the binding process occurred. After binding, antibodies against NF-κBp65 and NF-κBp50 were pipetted, followed by the secondary antibodies and a developing solution. Absorbance was then measured and compared between the groups.

### 2.6. Statistical Analysis

**Figure 1:**
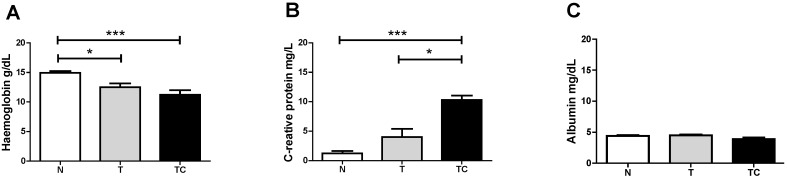
Serum Hemoglobin (**A**) C-Reactive Protein (**B**) and Albumin (**C**) concentration. Data expressed as mean ± standard error; *: *p* < 0.05; ***: *p* < 0.001.

**Figure 2:**
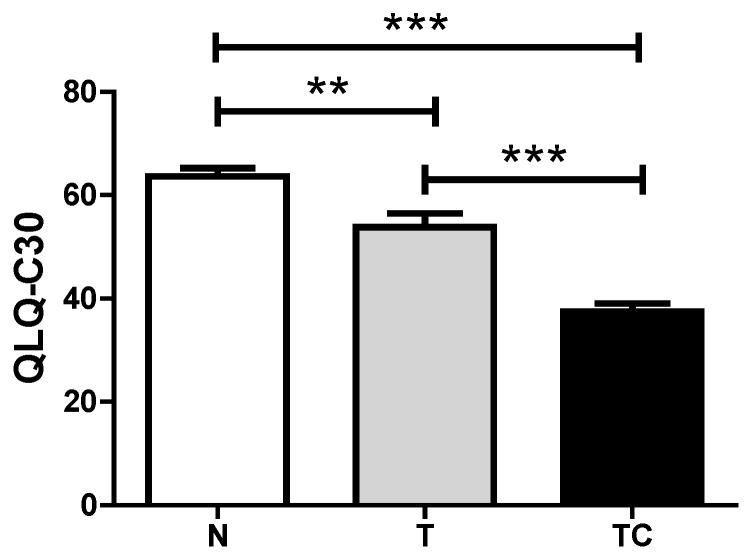
Quality of life Score. Data expressed as mean ± standard error; **: *p* < 0.01; ***: *p* < 0.001.

**Figure 3:**
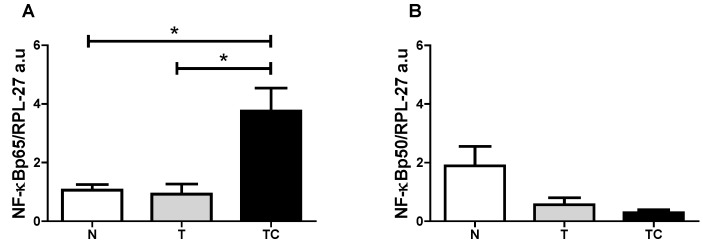
Subcutaneous adipose tissue NF-κBp65 and NF-κBp50 gene expression. (**A**) NF-κBp65/RPL-27 gene expression; (**B**) NF-κBp50/RPL-27 gene expression. Data expressed as mean ± standard error; *: *p* < 0.05.

General characteristics (Table 2), Biochemical parameters results are expressed as means ± SD (Figure 1), Quality of life Score (Figure 2) and gene expression (Table 3 and Figure 3) data are expressed as means ± SE.. Binding assay results are expressed as means ± SE of percentage of the control group value (Figure A2). Statistical significance was determined either by ANOVA non-parametric analysis (Kruskal-Wallis test with Dunn’s post-test), for those parameters that did not present equal variances, or ANOVA one-way with Tukey’s post-test, for the parameters that showed equal variance, as assessed by the Bartlett’s test. *p* < 0.05 was considered statistically significant. Spearman’s correlation analysis was then performed between paired samples. All statistics analyses were performed with the Graphpad Prism software (version 5.0).

**Table 2:** General characteristics of patients in each group.

	*N*	T	TC	*p*
***n***	12	11	12	
**Male/Female (*n*)**	9/3	7/4	6/6	
**Age (years)**	62.00 ± 2.51	58.64 ± 4.04	60.42 ± 2.93	0.7609
**Height (m)**	1.65 ± 0.03	1.64 ± 0.02	1.64 ± 0.02	0.9909
**Previous body mass (Kg)**	75.48 ± 4.86	75.64 ± 4.532	74.44 ± 2.665	0.9728
**Current body mass (Kg)**	75.48 ± 4.86	67.83 ± 3.87	64.45 ± 2.98	0.1432
**Δ Body mass (%)**	0.00 ± 0.00	9.36 ± 3.27 *	13.58 ± 1.75 *	**0.0005**
**BMI (kg/m^2^)**	27.76 ± 1.40	25.31 ± 1.58	23.89 ± 1.16	0.1573
**Tumor stage**				
**I**	-	18.2%	0%	-
**IIA/IIB/IIC**	-	27.3%	25%	-
**IIIA/IIIB/IIIC**	-	45.4%	33.3%	-
**IVA/IVB**	-	9.1%	41.7%	-
**Primary tumour site**				
**Colon and rectum**	-	72.7%	58.3%	-
**Stomach**	-	18.2%	41.7%	-
**Other**	-	9.1%	0%	-

Data expressed as mean ± standard error. Δ: Difference between self-declared previous body mass and current body mass. *: Significant difference *versus* N.

**Table 3:** Subcutaneous adipose tissue NF-κB signaling pathway proteins and pro-inflammatory mediators under NF-κB control gene expression.

Gene Expression	Statistical Analysis	Significance
**(A) NF-κBp65**	*p* = 0.0147	TC *vs.* T; TC *vs.* N
**(B) NF-κBp50**	*p* = 0.1719	---
**(C) IL-6**	*p* = 0.1458	---
**(D) IL-1β**	*p* = 0.0049	TC *vs.* T
**(E) TNF-α**	*p* = 0.0201	TC *vs.* N
**(F) INF-γ**	*p* = 0.2255	---
**(G) MCP-1**	*p* = 0.0033	TC *vs.* T; TC *vs.* N
**(H) IkB-α**	*p* = 0.0019	TC *vs.* T; TC *vs.* N

(**A**) Gene expression analysis of NF-κBp65 showed higher values (*p* = 0.0147) in cachectic cancer patients compared to controls; (**B**) Gene expression of NF-κBp50 protein showed no differences among the patients (*p* = 0.1719); (**C**) IL-6 gene expression showed no differences among the patients (*p* = 0.1458); (**D**) IL-1β gene expression was higher in cachectic cancer patients (*p* = 0.049) compared to non-cachectic patients; (**E**) TNF-α gene expression was higher in cachectic cancer patients (*p* = 0.0201) compared to the control group; (**F**) INF-γ gene expression showed no differences among the groups (*p* = 0.2255); (**G**) MCP-1 gene expression was higher in cachectic cancer patients (*p* = 0.0033), compared to controls; (**H**) The inhibitory protein IκB-α gene expression was higher in cachectic cancer patients (*p* = 0.0019), compared to controls.

## 3. Results

### 3.1. Clinical Findings

Baseline characteristics of the patients are shown in Table 2. The subjects in the three groups were of similar height, weight and body mass index (BMI). Non-cachectic and cachectic cancer patients showed a significant difference in serum hemoglobin (*p* < 0.001), compared with the control group. TCC also presented significantly higher CRP serum levels (*p* < 0.001) compared with the other groups. We did not evaluate lean body mass among the groups, although groups were matched by BMI.

### 3.2. Quality of Life Analysis

The three parameters that compose the criteria for global quality of life analysis showed cachexia to negatively influence these parameters (*p* < 0.001). Non-cachectic cancer patients also demonstrated a reduction in quality of life compared with the control group (*p* < 0.001), as shown in Figure 2.

**Figure 4:**
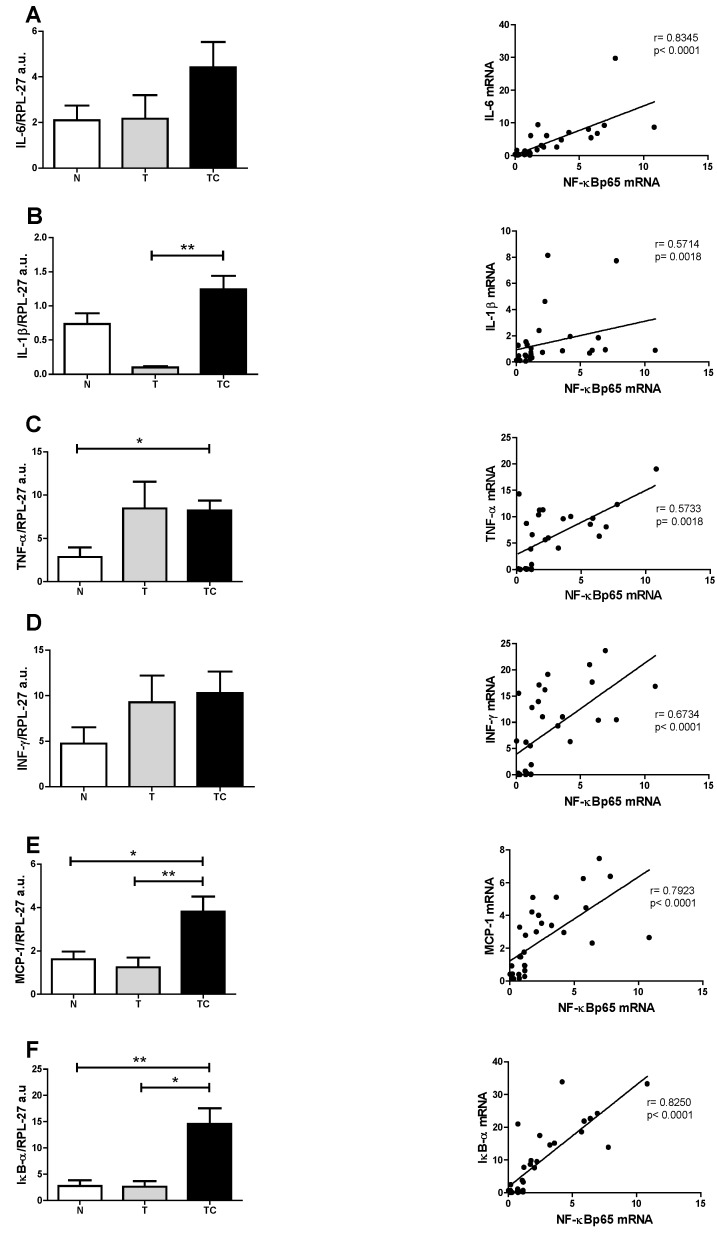
Subcutaneous adipose tissue gene expression and Spearman’s correlation with NF-κBp65. (**A**) IL-6; (**B**) IL1-β; (**C**) TNF-α; (**D**) INF-γ; (**E**) MCP-1; (**F**) IκB-α. Data expressed as mean ± standard error. *: *p* < 0.05; **: *p* < 0.01.

### 3.3. Gene Expression

Groups: Control (N), non-cachectic cancer patients (T) and cachectic cancer patients [16].

## 4. Discussion

**Figure A1:**
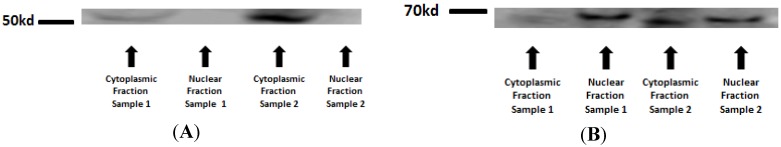
Western blot for nuclear and cytoplasmic protein extraction purity confirmation: β-Tubulin—51kd (**A**) and Lamin A—69 kd (**B**).

**Figure A2:**
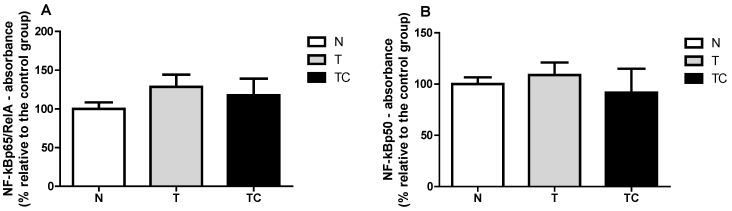
NF-κBp65 binding to the DNA NF-κB promoter region (**A**) and NF-κBp50 binding to the DNA NF-κB promoter region (**B**).

Systemic inflammation is a central feature of cancer cachexia [16,20,21,22]. Circulating pro-inflammatory mediators such as Il-6, TNF-α and acute phase proteins (CRP) are upregulated in cachectic patients and correlate positively with weight loss and poor prognosis. In this study, CRP concentration was significantly higher in cachectic cancer patients. This acute-phase protein has been described as a marker of systemic inflammation and is also considered as part of cachexia diagnostic criteria [16]. Our results reinforce the importance of circulatory pro-inflammatory mediators as cachectic markers and corroborate previous studies with cancer patients [23]. The search for non-invasive cachexia markers is mandatory, and would warrant earlier intervention, thus preventing the onset of the symptoms and adverse prognosis. In this study, quality of life was evaluated by the application of the QLQ-C30 questionnaire. Patients reported diminished quality of life, which compromises the treatment adherence and survival. The three clusters analyzed in this questionnaire: Functionality (physical, cognitive, emotional and social), Symptomatic (fatigue, pain, nausea and vomiting) and Global health were all impaired in the cachectic cancer patients. Amongst the symptomatic cluster, fatigue is the main declared symptom and is usually present in more than 75% of cachectic patients [24]. It is accepted that systemic inflammation markedly contributes to the worsening of cachexia prognosis. Several peripheral organs suffer the consequences of inflammation triggered by the high circulatory level of pro-inflammatory mediators, that, in turn, induce in peripheral and central organs the activation of the inflammatory signaling pathways, such as the NF-κB pathway [25]. The NF-κB/Rel family of proteins, described first by Sen and Baltimore in 1986 [26] consists of transcription factors intensely studied due to the major implication as key mediators of a wide variety of cellular responses associated mainly with inflammation, infection and apoptosis. These include the stimulation of the expression of pro-inflammatory mediators such as TNF-α, IL-6, INF-δ, IL-1β, of chemokines such as MCP-1, and of reactive oxygen species (ROS) [12,13,27]. The relationship of the NF-κB signaling pathway with poor prognosis of cancer cachexia has already been extensively studied in the muscle, where increased NF-κB signaling has been described in patients [28]. Similarly, in patients under treatment for lung cancer, circulating pro-inflammatory mediators were associated with the activation of the NF-κB signaling pathway in the muscle [29]. Moreover, the importance of this transcription factor was confirmed by pharmacological inhibition, which was demonstrated to be an effective tool to reduce muscle proteolysis and consequently, atrophy [30,31]. Besides the muscle, other organs such as the liver, the brain, the gut and the adipose tissue are affected by cachexia. Adipose tissue metabolism is impaired and extensive lipolysis is observed [32]. WAT actively contributes to the inflammatory state in cachexia by actively secreting pro-inflammatory mediators [4,6,33]. Despite being recognized as an active player in cachexia, no information is available in the literature about the role of NF-κB in the development and maintenance of local inflammation of the adipose tissue. In the present study we demonstrate for the first time, that gene expression of NF-κBp65, which is a subunit of the one of the most important transcription factors that induce pro-inflammatory mediator gene expression, is upregulated in the subcutaneous adipose tissue of cachectic cancer patients, compared with controls. This is a strong indication of the role of NF-κBp65 in the promotion of inflammation in the subcutaneous adipose tissue in cachectic cancer patients. To confirm such participation of NF-κBp65 in the regulation of the adipose tissue inflammation, an assay was performed to evaluate NF-κBp65 and NF-κBp50 binding capacity to its promoter region of the DNA. This assay may be envisaged as a ‘snapshot’ of the cellular nucleus subunits NF-κBp65 and NF-κBp50 capacity of action, at the moment of tissue collection. Previous experiments of our group showed that the NF-κBp65 binding to its promoter region is higher, but not statistically significant among groups, while NF-κBp50 did not differ among groups (Figure A1). This assay, nevertheless, does not provide optimal evidence of total binding rate, as NF-κB dimer activity presents a fast up-regulation in the nucleus and then decays rapidly, migrating back to the cytoplasm, where it is again sequestered by its inhibitory protein, IκB-α. This is described as a rapid and dose-dependent response, that involves phosphorylation and subsequently proteasome degradation of the NF-κB inhibitory protein, IκB-α, which leaves the transactivation domain of the NF-κB dimer free to translocate to the nucleus and exert its functions as a transcription factor [34]. This rapid and transient stimulus, however, is sufficient to alter gene expression and cause prolonged changes in the NF-κB target proteins mRNA levels [35]. This led us to hypothesize that actually the best experiment to examine NF-κBp65 activity would rather consist of sequential evaluation of binding at different collection times; what is, unfortunately not possible in a human study due to ethical limitations. An alternative, however, would be the analysis of the expression of its target genes. Thus, we proceeded with the gene expression analysis of the NF-κBp65 pro-inflammatory target genes by qPCR, having found that IL-1β, TNF-α and MCP-1 expression were upregulated in cachectic cancer patients compared with controls. This is a strong indication that the local subcutaneous adipose tissue inflammation described in cachectic cancer patients may be mediated by the increased expression and activity of NF-κBp65. Considering that several inflammatory signaling pathways work in concert in promoting inflammation and, in order to verify the specific relationship between NF-κBp65 and the induction of pro-inflammatory genes expression, we performed Spearman’s correlation tests. The results show that all genes described as NF-κBp65 targets present a positive correlation with the expression of NF-κBp65 in the patients’ subcutaneous adipose tissue, including its inhibitor IκB-α, which is significantly more expressed in cachectic cancer patients, as compared with controls (Figure 4). These data strongly corroborate the NF-κB target gene expression results obtained in the study. Furthermore, NF-κBp65 is the most active protein able to induce IκB-α expression [36] and the accumulation of newly synthesized IκB-α is described as a pivotal factor in the termination of NF-κB activity and shuttling NF-κB complexes from the nucleus back to the cytoplasm [36]. Therefore, parallelism between NF-κBp65 and IκB-α, in a feedback mechanism was expected. This result was a confirmation of previous studies that pointed out a role of the NF-κB signaling pathway in the promotion of an inflammatory state related with cachexia. Despite these very encouraging results, other signaling pathways such as the signal transducer and activator of transcription 3 (STAT3), the c-Jun N-terminal kinase (JNK), p38 and AP-1 are also of interest in WAT inflammation. While in this study we focused solely on the NF-κB signaling pathway, these other pro-inflammatory pathways may not be disregarded, as they are known to participate actively as co-players of the worsened prognosis in cachexia [37,38,39]. The limitations of the study should be acknowledged. There was no measurement of the patient’s lean body mass, although the patients and groups presented similar BMI. Owing to human tissue sample implicit variation, some of the analyses were not performed with the total number of patients formerly enrolled as some samples fell out of the detection range of assays.

## 5. Conclusions

NF-κB classical signaling pathway protein NF-κBp65 gene expression is increased in the subcutaneous white adipose tissue of cachectic cancer patients. Its target genes IL-1β, TNF-α, MCP-1 and IκB-α are also up-regulated. NF-κBp65 gene expression was positively correlated with all the targets genes analysed in this study. We strongly suggest a role for the NF-κB classical pathway in the inflammation of WAT in cachectic cancer patients.

## Appendix

### NF-κB Binding Assay

Groups: Control (N), non-cachectic cancer patients (T) and cachectic cancer patients [16].

In order to assure that only nuclear proteins were used in the assay, first a Western blot assay with antibodies against a specific nuclear protein (Lamin A) cytoplasmic protein (β-Tubulin) in two different samples (sample 1 and 2) was performed. The cytoplasmic fraction was contaminated with nuclear proteins, but the nuclear fraction, which was employed in the assay, was free from cytoplasmic protein. The binding assay revealed no difference between the groups in the two NF-κB subunits: NF-κBp65 (*p* = 0.2874) and NF-κBp50 (*p* = 0.1469).
